# Addressing Acid-Catalyzed Deamidation and the Solubility of Hydrophobic Peptides in Multi-Attribute Method Workflows

**DOI:** 10.1021/acs.analchem.3c02609

**Published:** 2023-10-12

**Authors:** Dan B. Kristensen, Martin Ørgaard, Trine M. Sloth, Gerard Comamala, Pernille F. Jensen

**Affiliations:** Symphogen, Pederstrupvej 93, 2750 Ballerup, Denmark

## Abstract

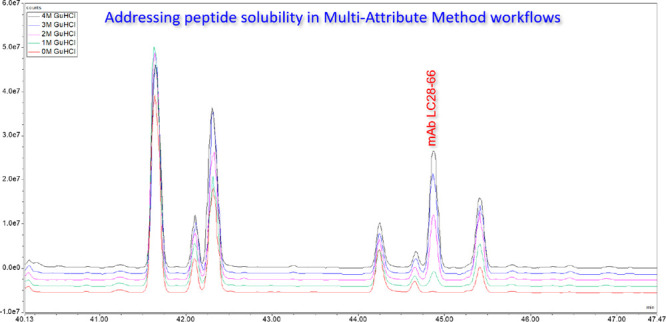

Recently, we introduced an optimized and automated Multi-Attribute Method (MAM) workflow, which (a) significantly reduces the number of missed cleavages using an automated two-step digestion procedure and (b) dramatically reduces chromatographic peak tailing and carryover of hydrophobic peptides by implementing less retentive reversed-phase column chemistries. Here, further insights are provided on the impact of postdigest acidification and the importance of maintaining hydrophobic peptides in solution using strong chaotropic agents after digestion. We demonstrate how oxidation can significantly increase the solubility of hydrophobic peptides, a fact that can have a profound impact on quantitation of oxidation levels if care is not taken in MAM workflows. We conclude that (a) postdigestion acidification can result in significant acid-catalyzed deamidation during storage in an autosampler at 5 °C and (b) a strong chaotropic agent, such as guanidine hydrochloride, is critical for preventing loss of hydrophobic peptides through adsorption, which can result in (sometimes extreme) biases in quantitation of tryptophan oxidation levels. An optimized method is presented, which effectively addressed acid-catalyzed deamidation and solubility of hydrophobic peptides in MAM workflows.

Peptide mapping by LC-MS, also referred to as the Multi-Attribute Method (MAM), is a well-established technology for the characterization of biopharmaceutical product quality.^1−3^ It is routinely used for the verification of the primary structure as well as a site-specific, quantitative evaluation of critical post-translational modifications, or quality attributes of biopharmaceutical products.^4−7^ The information provided by MS-based peptide mapping supports biopharmaceutical development across all development stages, ranging from lead selection, developability assessment, comparability studies, process support, and general product characterization.^8−11^ Among 80 Biologics License Applications (BLAs) submitted between 2000 and 2015, 79 were found to use MS to support product characterization.^12^ MAM is currently applied primarily as a characterization tool to support monitoring of critical quality attributes (CQAs) and to detect new peak intensity during biopharmaceutical development. However, in recent years MAM has advanced into current Good Manufacturing Practice (cGMP) environments, where it is used for release and stability testing of biopharmaceuticals.^2,7,13^ Indeed, MAM has now been implemented for cGMP testing of some biopharmaceutical products, replacing several conventional impurity assays.^3,13,14^ With the growing importance and adaptation of MAM, an industry-wide consortium has been formed to facilitate knowledge sharing on MS-related assays among the biopharma companies, technology providers, and regulatory agencies (www.mamconsortium.org).

Recently, we introduced an automated MAM workflow that significantly reduces the number of missed cleavages by performing digestion in two independent steps at high (75 °C) and low (40 °C) temperature using a benchtop robot and trypsin immobilized to magnetic beads.^15^ In addition, the optimized MAM workflow radically reduces chromatographic peak tailing and carryover of hydrophobic peptides by switching from traditional C18-based reversed-phase (RP) column chemistry to less retentive C4 column chemistries.^15^ Often, complementarity-determining regions (CDRs) of mAbs are rich in aromatic amino acids, resulting in highly hydrophobic peptides after trypsin digestion, which are challenging to analyze by traditional C18 column chemistries. The switch to RP column chemistries of lower retentivity was found to impact MAM data quality and robustness for these critical, CDR spanning peptides in a profoundly positive manner.^15^

In the current technical note, we evaluate the impact of the post digestion acidification step commonly used in MAM workflows, as well as the importance of adding solubilizing chaotropic reagents, such as guanidine hydrochloride (GuHCl), to prevent loss of hydrophobic peptides after digestion through adsorption to the walls of the digestion well.

Post digestion acidification is commonly used in peptide mapping workflows using in-solution digestion to (1) inactivate enzymatic activity, (2) to prevent peptide deamidation, i.e., chemical stabilization of peptides, and (3) adjust pH for analysis.^16,17^ Evolution of protein digestion workflows has to a large extent been driven by proteomic communities looking to simplify, accelerate, and automate the digestion procedures, with the aim of identifying and quantitating a large number of proteins and/or post-translational modifications controlling cellular activity (e.g., phosphorylation in signaling pathways) typically from limited samples.^17^ Chemical degradation of proteins, such as deamidation, is not *per se* a focus area of proteomics, and as such, detailed focus has not been on avoiding these modifications during sample preparation. One example of a commonly used legacy step is the acidification of samples after proteolytic digestion, to inactive protease and prevent high pH-induced deamidation, the latter being a key focus area of MAM communities.^16,17^ Typically from 0.1 to 2% trifluoroacetic acid (TFA) is used for acidification in MAM workflows.^13,15,18,19^ At neutral and basic pH, Asn deamidation proceeds through a succinimide intermediate to form Asp and isoAsp, a well known and major degradation pathway for many biopharmaceutical proteins and a typical focus area in MAM sample preparation workflows.^20,21^ However, at low pH (<3), deamidation can occur through direct acid-catalyzed hydrolysis to form Asp only, so excessive acidification can potentially be an issue in MAM workflows.^20,22^ In our laboratory, observations indicated potential deamidation stability issues with acidified samples after prolonged storage in an autosampler (unpublished observations). For the above reasons and because our current digestion workflow employs an enzyme immobilized on magnetic beads, which are removed after digestion (i.e., no enzyme inactivation is required after digestion), we decided to do an in-depth evaluation of the impact and necessity of the post-digest acidification step. Focus was on the impact of acidification using 1% TFA, as described in our original publication, as well as the original MAM publication by Rogers et al.^13,15^

Trypsin digestion of antibodies and derived formats often result in a subset of hydrophobic peptides spanning critical CDR regions, which may perform poorly on traditional C18 RP column chemistries and which may be challenging to keep in solution in aqueous solvents.^15^ We addressed poor chromatographic performance and poor solubility by switching to RP column chemistries with lower retentivity (C4) and by manually adding 2 M GuHCl to the samples after digestion, respectively.^15^ In the current manuscript we further improve the original two-step MAM workflow by including an automatic GuHCl wash step that is performed directly in the 96-deep-well plates by the employed robot as part of the automatic digestion program. This automatic GuHCl wash step ensures that all surfaces the sample is exposed to during digestion are exposed to high levels of GuHCl, ensuring that sticky, hydrophobic peptides remain in solution for subsequent LC-MS/MS analysis. Surprisingly, it is difficult to find scientific literature addressing the challenge of hydrophobic peptides and their solubility in the context of MAM workflows. Most established MAM workflows use denaturing/solubilizing reagents (e.g., urea, GuHCl) for protein denaturation, reduction, and alkylation, but typically remove these reagents by buffer exchange prior to digestion, thus leaving the resulting peptides in an aqueous environment.^16,17^ In the current technical note, we evaluate the correlation between GuHCl concentration, peptide solubility, and quantitation biases relating to different solubilities of hydrophobic peptides and their oxidized counterparts. It is demonstrated that a strong chaotropic agent, such as GuHCl, is required for keeping some hydrophobic peptides in solution and for preventing (sometimes extreme) biases in quantitative results for oxidations in highly hydrophobic peptides. The updated MAM workflow presented here effectively addresses challenges relating to the solubility of hydrophobic peptides and their oxidized counterparts.

## Experimental Section

All experiments were performed as described by Kristensen et al., with the exceptions described below.^15^

### Chemical and Reagents

mAbs were produced internally at Symphogen. Thermo Scientific SMART Digest kits and associated low pH digestion buffer were obtained from Thermo Fisher Scientific. UHPLC MS-grade water and acetonitrile were purchased from Fisher Scientific. MS grade (IonHance) difluoroacetic acid (DFA) was purchased from Waters. 0.5 M tris(2-carboxyethyl)phosphine hydrochloride (TCEP) and 8 M guanidine-HCl (GuHCl) were obtained from Pierce. RAPID Slit Seals were obtained from BioChromato.

### Analytical Instrumentation and LC-MS Data Management

LC-MS/MS was performed using a Thermo Scientific Vanquish Horizon UHPLC coupled to a Thermo Scientific Orbitrap Fusion Tribrid MS equipped with an Ion Max source and the HESI-II-probe as described previously.^15^ All data was acquired using Thermo Scientific Chromeleon CDS Enterprise version 7.3.1. LC-MS/MS data stored in Chromeleon CDS were automatically synchronized to Protein Metrics Byosphere Enterprise. All MS data processing was performed in Byosphere Enterprise.

### SMART Digestion Protocol

Samples were digested in a Thermo Scientific KingFisher Duo Prime robot using Thermo Scientific SMART Digest Trypsin using Thermo Scientific BindItTM software version 4.0 (see Figure S1 for details). Samples were mixed with SMART digestion buffer (pH 6.5) and TCEP in row A of a KingFisher 96 deep well plate. Final sample and TCEP concentration were 1 mg/mL and 5 mM, respectively. Final digestion volume was 100 μL. The KingFisher Duo digest program consisted of the following steps:

1. Collect first SMART Digest trypsin resin (15 μL resin in 100 μL digestion buffer, row C) and wash in 200 μL SMART Digest buffer (row E).
2. Digest for 15 min at 75 °C (row A).
3. NEW STEP: Collect first SMART Digest trypsin resin from row A after digestion, wash in 100 μL GuHCl solution (row G), collect and discard in waste lane (200 μL digest buffer, row F).
4. Collect second SMART Digest trypsin resin (15 μL resin in 100 μL digestion buffer, row D) and wash in 200 μL SMART Digest buffer (row E).
5. Digest for 30 min at 40 °C (row A).
6. NEW STEP: Collect second SMART Digest trypsin resin from row A after digestion, wash in 100 μL GuHCl solution (row G), collect and discard in waste lane (200 μL digest buffer, row F).
7. KingFisher Duo digest program complete.

The automatic wash steps in GuHCl after each digestion steps differ from the original publication, in which GuHCl was manually added to the sample lane (row A) after digestion.^15^ The automatic GuHCl wash step ensures that all surfaces the sample is exposed to (sample well, SMART Digest resin, and KingFisher plastic comb) are exposed to GuHCl. The GuHCl concentration varied from 0 to 8 M in the current study. SMART Digestion buffer was used for dilution of the 8 M GuHCl commercial stock solution. After digestion, the GuHCl wash solutions (row G) were transferred to the samples (row A). Samples were either analyzed without acidification (pH 6.5) or acidified by adding 20% TFA to a final concentration of 1% (pH 0.5).^13,15^ In addition, acidification was performed by adding 20% formic acid, 20% TFA, or 20% DFA to a final concentration of 1% (pH 2.3), 0.1% (pH 5.6), or 0.1% (pH 5.3), respectively. The 96-deep-well plates were covered with a Rapid Slit Seal, mixed by vortexing (1400 rpm, 10 s) using a ThermoMixer and transferred to the Vanquish autosampler for LC-MS/MS analysis.

### LC-MS/MS Analysis

Solvent A was 0.1% DFA in water. Solvent B was 95% acetonitrile with 5% water and 0.1% DFA. The LC method includes a gradient from 2 to 45% solvent B from 1 to 52 min and two wash steps, and this method was used throughout (see Figure S2 for details).^15^ Total run time was 70 min and column temperature was 25 °C. Flow rate was 0.4 mL/min, and 8 μg sample load was used throughout. All samples were analyzed using Thermo Scientific Hypersil C4 GOLD columns (2.1 mm × 150 mm, 1.9 μm particles).

LC-MS/MS was performed using data-dependent acquisition (DDA) on an Orbitrap Fusion. Basically, MS was performed in the Orbitrap detector and MS/MS (EThcD and HCD) in the ion trap detector, and precursors were excluded for 7 s (see Figure S3 for details). Flow was diverted from waste to MS at 1.2 min (was 3 min in the original manuscript) after injection, and back to waste after 69 min. The flow path was changed using the postcolumn switching valve of the Vanquish column compartment. Since the original publication, it was discovered that several hydrophilic, small peptides elute in the 1.3 to 3 min range, so time to waste was changed to 1.2 min in the current workflow.^15^ This generally ensures >99% sequence coverage in the updated workflow, compared to >96% in the original workflow (data not shown).

### Data Processing

All LC-MS/MS was processed in Byosphere Enterprise using the Byosphere Client Version 5.0. Since no alkylation step is required in the SMART Digest workflow, cysteine residues are searched as reduced forms.

## Results and Discussion

This section is divided into two parts covering (1) the impact of post-digestion acidification and (2) the impact of post-digestion addition of GuHCl, respectively.

### Critical Look at Post-Digestion Acidification

**Figure 1:**
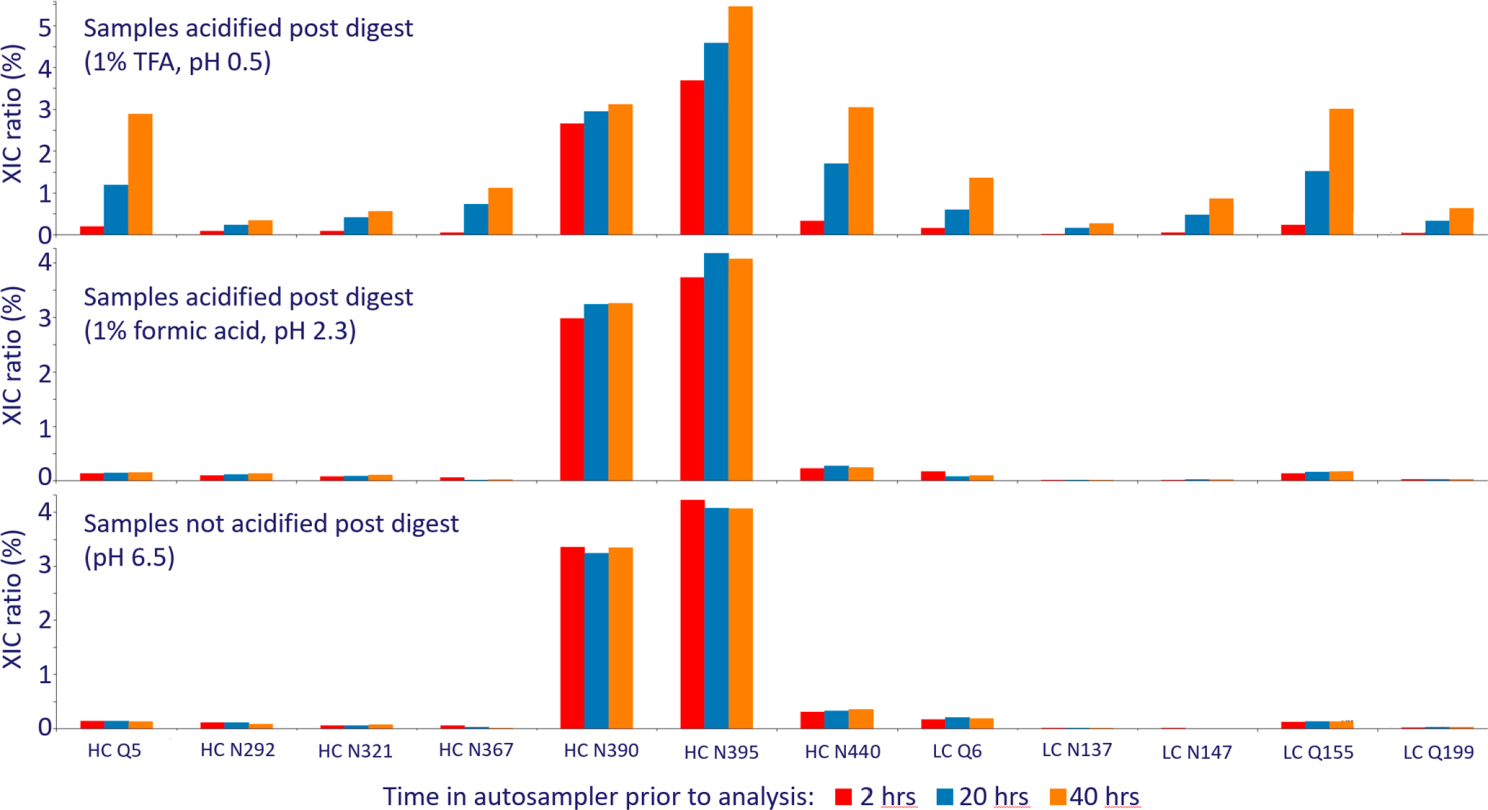
Deamidation levels observed for an internal Symphogen mAb after 1 year at 25 °C. Sample were either acidified with 1% TFA (pH 0.5), 1% formic acid (pH 2.3), or not acidified (pH 6.5). Samples were analyzed repeatedly for up to 40 h after digestion (stored at 5 °C in autosampler).

Between 0.1 and 2% TFA is routinely used for acidification after digestion in MAM workflows.^13,15,18,19^ Avoidance of high pH-catalyzed deamidation is a focus area the MAM community often use to support the use of a postdigest acidification step. However, deamidation may also take place at low pH, a topic much less covered in the MAM literature.^20,22^ We investigated the in-use sample stability (up to 40 h in autosampler at 5 °C) after acidification with 1% TFA as described previously, as well as acidification with 1% formic acid or no acidification (pH 6.5). Figure 1 clearly illustrates that acid-catalyzed deamidation takes place in an autosampler after acidification with 1% TFA. Indeed, many of the identified deamidation sites in TFA-acidified samples are missing or very low level in the nonacidified or formic-acid-acidified samples.

A distinct time-dependent rise in deamidation is observed for samples acidified with 1% TFA, confirming that acid-catalyzed deamidation is taking place in the autosampler at a significant rate and short time scale (hours or days). Acid-catalyzed deamidation is nearly absent in formic-acid-acidified samples, and no increase in deamidation is observed for nonacidified samples stored at pH 6.5. Main deamidation sites are in the well-known PENNY peptide (HC N390 and N395). Our results clearly demonstrate that harsh acidification, such as 1% TFA, will induce acid catalyzed deamidation and should be avoided. If acidification is required, it is recommended to use milder conditions, such as 1% formic acid, 0.1% TFA or 0.1% DFA, which does not result in acid induced deamidation during storage in autosampler (see Figure S4). In our MAM workflow, the acidification step can be removed all together since the immobilized SMART Digest enzyme is removed after digestion by the robot and since deamidation levels are stable at pH 6.5, which is the pH of the SMART Digest buffer, when stored in the autosampler. According to the literature deamidation rates are minimal in the pH 3–4 range, and this can be considered as part of the post-digestion acidification procedure.^22^ Oxidation levels were also stable in the autosampler without post-digested acidification (data not shown). No disulfide linkages are reformed with the updated workflow (data not shown), which is expected since TCEP is present in the samples until the LC MS analysis and since LC separation is performed under acidic conditions (0.1% DFA), where thiol groups are protonated and nonreactive.

**Figure 2:**
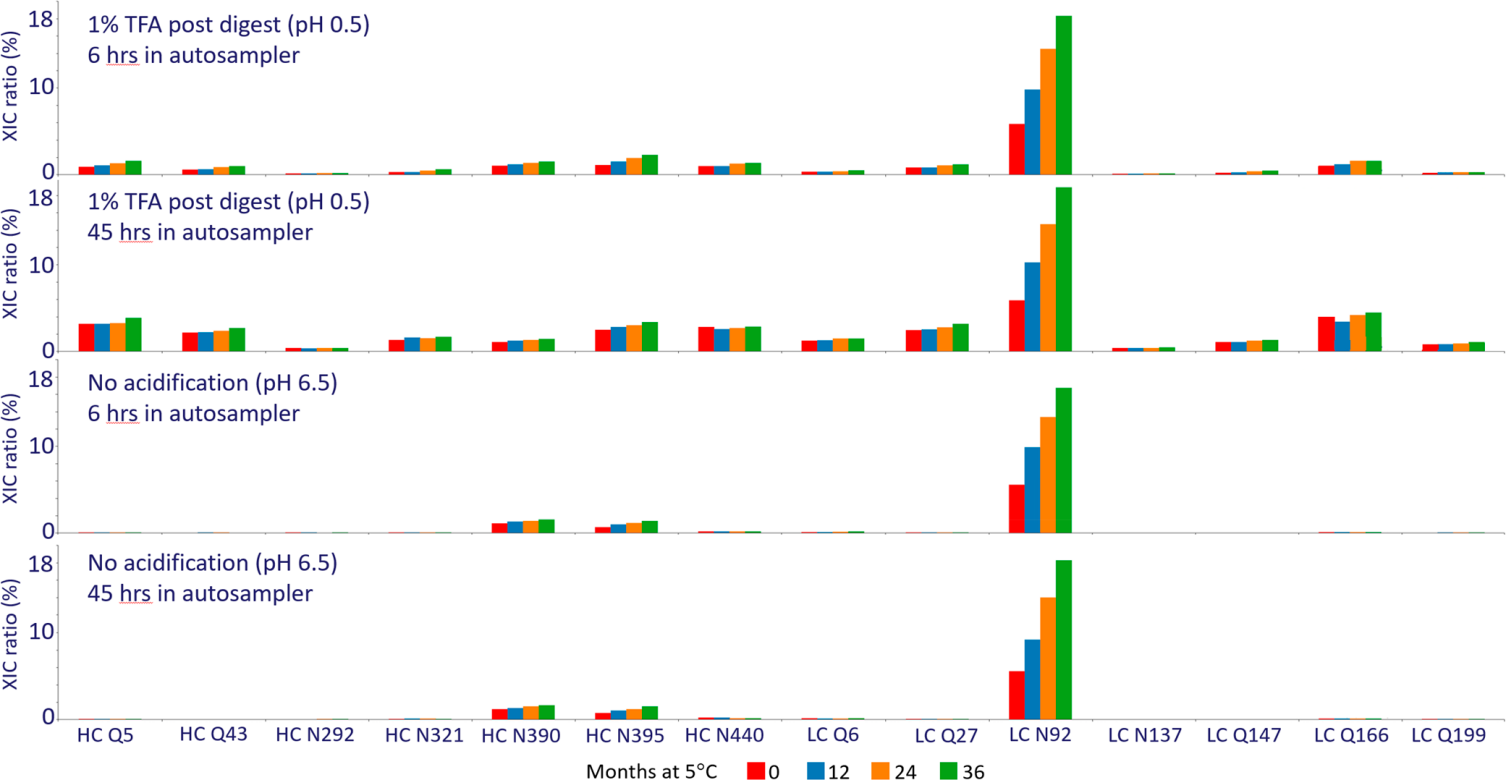
Deamidation levels from a stability study of internal mAb. Drug product was stored at 5 °C for three years and samples were collected annually. Samples were digested according to the current MAM workflow. After digestion the samples were either not acidified (pH 6.5) or acidified with 1% TFA (pH 0.5) and analyzed repeatedly by LC-MS/MS. Deamidation levels are shown after the 1st run (average of 6 h in autosampler) and after the 5th run (average of 45 h in the autosampler).

Next, deamidation levels were investigated for a stability study performed on an internal project at Symphogen. The stability samples were analyzed without acidification (pH 6.5) and with postdigested acidification (1% TFA). The four stability samples (0, 12, 24, 36 months at 5 °C) were analyzed repeatedly by LC-MS/MS, and results from the first run (average of 6 h in autosampler) and fifth run (average of 45 h in autosampler) are shown in Figure 2. The TFA-acidified samples display a distinct rise in artifactual acid-catalyzed deamidation with increasing time in autosampler. In contrast, the nonacidified samples show no artifactual acid induced deamidation, and results are consistent and independent of time in autosampler. Based on the nonacidified samples, it can be concluded with confidence that true deamidations, which increase in the stability study are well-known deamidations in the PENNY peptide (HC N390, HC N395) and finally LC N92, which is the major degradation pathway for this biopharmaceutical product. Again, 1% TFA results in acid-catalyzed deamidation during storage in the autosampler at 5 °C, leading to a high background in deamidation signals. In contrast, deamidation results are fully consistent in the nonacidified samples over time in the autosampler.

In conclusion, the new MAM digestion workflow, with no or mild acidification after digestion, effectively removes acid-catalyzed artifacts and provides a simpler, easier way to interpret results and pinpoint true deamidation from, e.g., stability studies.

### Linking Peptide Solubility, Oxidation, Quantitative Biases, and GuHCl Levels

**Figure 3:**
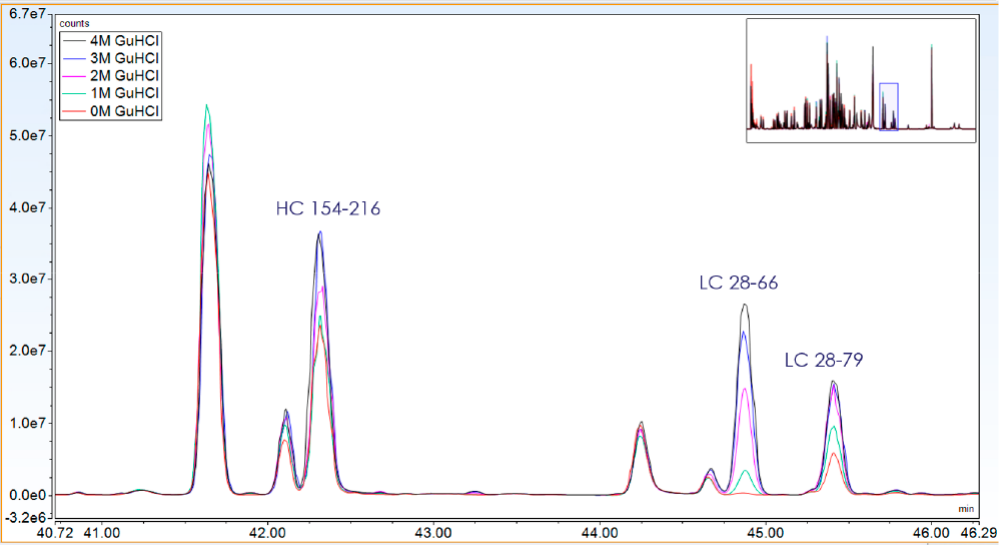
Zoomed base peak chromatograms (BPCs) of Symphogen internal mAb digested with trypsin. After digestion, the sample was mixed with 0, 1, 2, 3, or 4 M GuHCl and analyzed by LC-MS/MS within 8 h of digestion. A strong correlation is seen between the signal intensity and GuHCl concentration for some peptides, such as the LC28–66 peptide. Hydrophobic peptides, such as LC 28–66, therefore, rely heavily on GuHCl to stay in solution during LC-MS analysis. HC: heavy chain. LC: light chain. Numbers refer to amino acid residues.

Trypsin digestion of mAbs and other proteins often results in a subset of hydrophobic peptides that have poor solubility in aqueous solutions (Figure 3). For mAbs we typically observe that many of these hydrophobic peptides span the complementarity determining regions (CDRs), i.e., regions often rich in aromatic residues, which are critical for target binding and thus mAb function. Examples of such peptides are shown in Figure 3. In the absence of GuHCl the peptide spanning light chain (LC) 28–66 is nearly absent in the sample a few hours after digestion. Such peptides are easily recovered by adding GuHCl to the sample, confirming that these peptides adhere to the surface of the digestion well and have poor solubility in aqueous solvents.

**Figure 4:**
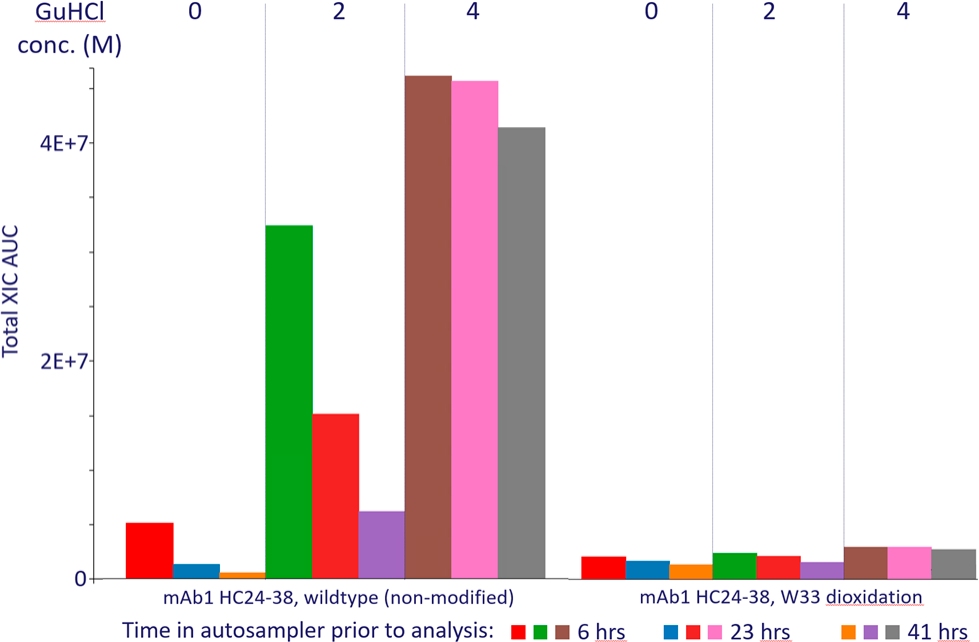
Total extracted ion current (XIC) for a nonmodified and oxidized peptide as a function of GuHCl concentration and time in the autosampler (5 °C). Each sample was injected 7 times, and results for run 1 (6 h), run 4 (23 h), and run 7 (41 h) are summarized here. The peptide (residues 24–38) spans CDR1 of the heavy chain. As the GuHCl concentration increases, the XIC increases radically for the nonmodified peptide and ultimately converges toward a stable level at 4 M GuHCl. This reflects the poor solubility of the wild-type HC 24–38 peptide in aqueous solvents (no chaotropic agent). In contrast, the oxidized version of the HC 24–38 peptide is significantly more soluble and constant over time in an aqueous solvent. The difference in solubility of nonmodified and oxidized peptides can lead to extreme quantitative biases when insufficient levels of GuHCl are used (Figure 5).

The loss of some hydrophobic peptides by adsorption into plastic in aqueous solvents has a profound impact. This observation should raise concerns about the quantitative accuracy of modifications present in these peptides. To evaluate this, we investigated the in-use stability (up to 41 h) of a tryptic digest of an internal mAb mixed with different levels of GuHCl concentrations added after digestion. Figure 4 illustrates the challenges associated with the poor solubility of some peptides in aqueous solvents. The wild-type peptide spanning CDR1 (residue 24–38) of the heavy chain is rich in aromatic residues and gives a weak signal intensity in aqueous conditions, which drops further over time (up to 41 h in autosampler) due to adsorption to the walls of the digestion well. High levels (4 M) GuHCl are required to keep the peptide in solution at a constant level over time. In contrast, and a very important observation, the oxidized form of peptide HC 24–38 shows good solubility and limited drop over time in aqueous solvent.

**Figure 5:**
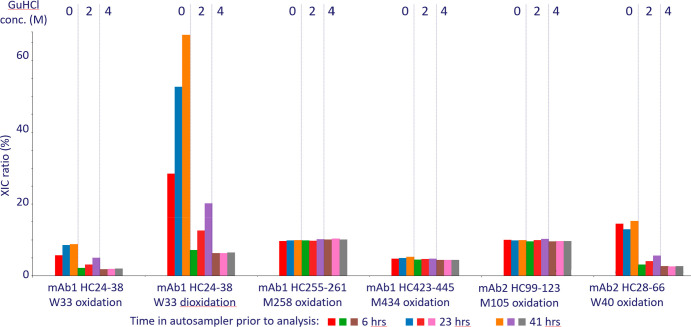
Oxidation levels for an internal project (mixture of two antibodies) measured as a function of GuHCl concentration and time in the autosampler (5 °C). Each sample was injected 7 times, and results for run 1 (6 h in autosampler), run 4 (23 h in autosampler), and run 7 (41 h in autosampler) are summarized here. For tryptophan oxidations, a strong impact is observed with respect to GuHCl concentration, reflecting different solubilities of nonmodified and oxidized peptides (Figure 4). For instance, at 0 M GuHCl mAb1 W33 dioxidation ranges from 28% to 67% after 6 and 41 h in the autosampler, respectively. As GuHCl concentration increases the mAb1 W33 dioxidation level converges to a time stable and constant level (6–7%).

The poor solubility and, in particular, the difference in solubility of nonmodified and oxidized peptides are quantitatively challenging in MAM workflows when insufficient levels of chaotropic agents are present. Figure 5 illustrates the importance of maintaining good and even peptide solubility when quantitating oxidation levels. At low GuHCl concentration, the poor solubility of nonmodified peptides spanning CDRs (mAb1 HC24–38, mAb2 HC 28–66) lead to an overestimation of W oxidation levels. The W oxidation levels drift to higher levels over time in the autosampler as more and more nonmodified peptides are lost by adsorption to the walls of the sample well. As the GuHCl concentration increases, the W oxidation converges to a time stable and constant level, since solubility issues are diminished by the GuHCl. In summary, GuHCl is critical for accurate quantitation of oxidation levels in hydrophobic peptides displaying poor solubility in aqueous solvents. The low solubility of some wild-type peptides in aqueous solvents correlates with a high content of aromatic amino acids, in particular, W residues. Consequently, the low solubility tends to correlate with increased retentivity on reversed-phase columns, since tryptophan is the strongest contributor to retentivity.^23^ Since tryptophan oxidation increases the hydrophilicity of a peptide, the quantitation bias described here is primarily associated with W oxidation and not M oxidation (see Figure 5). The reason for this is that there is no correlation between M content and peptide solubility in aqueous solvents (i.e., high M content does not decrease peptide solubility).

Based on the observations described above, it was decided to increase the GuHCl concentration from 2 to 4 M after digestion in the current, revised MAM workflow, in addition to implementing the automated GuHCl wash step, performed by the digestion robot.

It should be noted that low binding versions of 96-well plates have been evaluated previously, and these did not improve the peptide solubility issue (data not shown). Transfer to low-bind containers (e.g., glass) after digestion can also been considered. However, some hydrophobic peptides are lost by adsorption already during digestion, so it is important to recover these peptides in the original deep well plate rather than transferring samples to a new vessel. Finally, it is a priority to keep the current workflow automated, hands-off, and without any sample manipulation after digestion; i.e., the 96-deep-well plate used for digestion is transferred directly to the autosampler of the LC-MS system.

## Conclusion

Here we demonstrate that established post-digest acidification procedures using 1% TFA in MAM workflows can lead to significant acid-catalyzed deamidation during storage in an autosampler (5 °C) in a relatively short time frame (hours to days). Omitting the acidification step or using milder acidification (1% formic acid, 0.1% TFA or 0.1 DFA) reduces or eliminates acid-catalyzed artifacts and radically improves confidence and interpretation of deamidation results.

In addition, we demonstrate the importance of using strong chaotropic agents to address the low solubility of some hydrophobic tryptic peptides from mAb digests. It demonstrated how tryptophan oxidation can radically improve the solubility of a peptide relative to the nonmodified peptide, a fact that may result in extreme biases when quantitating oxidation levels in MAM workflows if care is not taken. By implementing an automatic wash step in GuHCl and increasing the final GuHCl concentration from 2 to 4 M after digestion, we effectively eliminate issues relating to the poor and differential solubility of hydrophobic peptides and their oxidized counterparts.

## Supporting Information Available

Additional information provided as noted in the text (PDF)
