# Supplementary material for: Addressing Acid-Catalyzed Deamidation and the Solubility of Hydrophobic Peptides in Multi-Attribute Method Workflows
Source: Anal Chem. 2023 Oct 12;95(42):15465–71. doi: 10.1021/acs.analchem.3c02609 (PMC10603607; doi:10.1021/acs.analchem.3c02609)
Supplement: Supplementary file 1 — ac3c02609_si_001.pdf [file ac3c02609_si_001.pdf]

SUPPORTING INFORMATION: ADDITIONAL EXPERIMENTAL DETAILS, MATERIALS, AND METHODS, INCLUDING PHOTOGRAPHS OF EXPERIMENTAL SETUP

ADDRESSING ACID CATALYZED DEAMIDATION AND SOLUBILITY OF HYDRO-PHOBIC PEPTIDES IN MUTI-ATTRIBUTE METHOD WORKFLOWS.

Dan B. Kristensen, Martin Ørgaard, Trine M. Sloth, Gerard Comamala, Pernille F. Jensen.

Symphogen, Pederstrupvej 93, 2750 Ballerup, Denmark.

TABLE OF CONTENT

FIGURES

Figure S1. KingFisher Duo Prime program used in the current study. 2

Figure S2. LC gradient used througout the current study for LC-MS analysis. Flow rates were 0.5 mL/min for Accucore columns and 0.4 mL/min for Hypersil GOLD columns. 2

Figure S3. Orbitrap Fusion MS method settings used in the current study. 3

Figure S4. Deamidation levels (extracted ion current ratio) was plotted against time (hrs) in auto sampler and linear regression was performed. The figure shows the determined slope for each deamidation site and acidification condition. A value close to zero indicates no increase in deamidation levels over time in the autosampler. A positive value indicates increasing deamidation level over time in the autosampler. Acidification using 1% TFA consistently gives a higher slope than other acidification conditions, confirming that acid-induced deamidation is taking place over time in the autosampler. H: heavy chain. L: light chain. Numbers refer to amino acid residues. 4

TABLES

Table S1. LC & MS Source Settings for Accucore and Hypersil GOLD columns 3

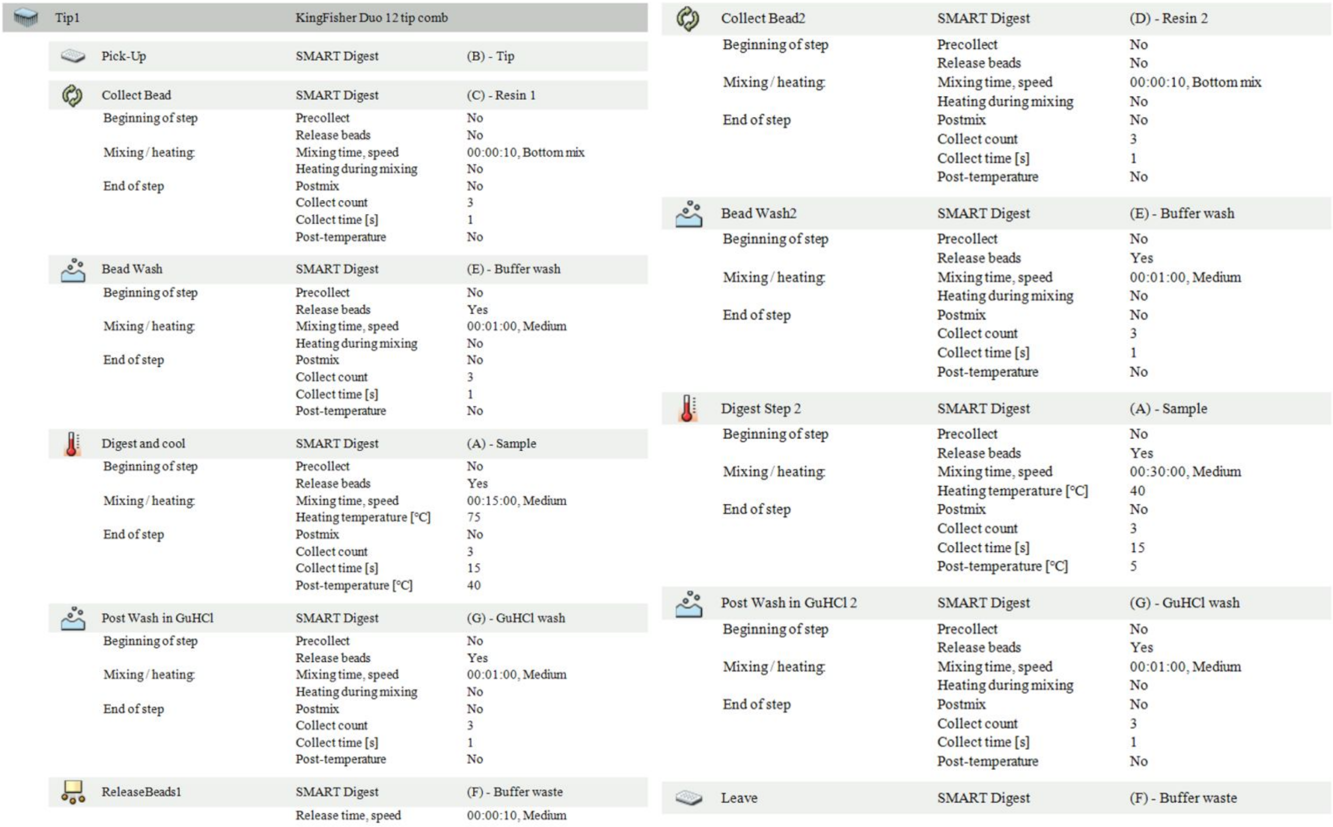

Figure S1. KingFisher Duo Prime program used in the current study.

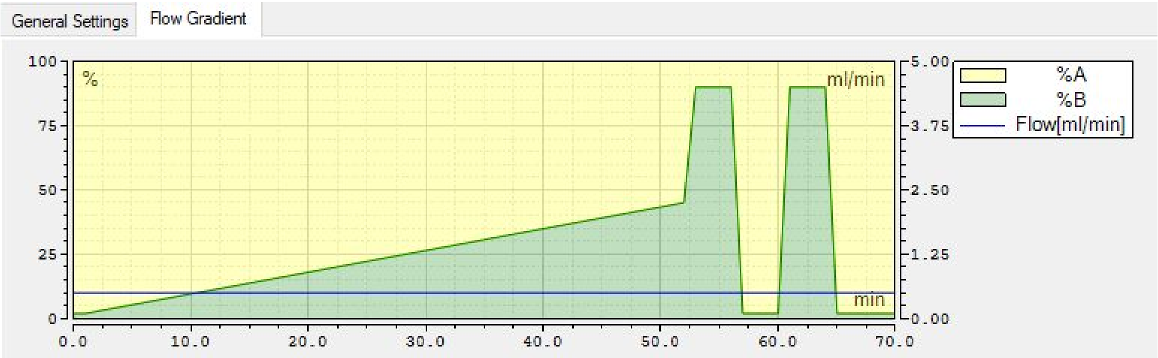

No	Time	Flow [ml/min]	%B	Curve
1	0.000		Run	
2	0.000	0.500	2.0	5
3	1.000	0.500	2.0	5
4	52.000	0.500	45.0	5
5	53.000	0.500	90.0	5
6	56.000	0.500	90.0	5
7	57.000	0.500	2.0	5
8	60.000	0.500	2.0	5
9	61.000	0.500	90.0	5
10	64.000	0.500	90.0	5
11	65.000	0.500	2.0	5
12	*New Row*			
13	70.000		Stop Run	

Insert

Delete

Figure S2. LC gradient used througout the current study for LC-MS analysis. Flow rates were 0.5 mL/min for Accucore columns and 0.4 mL/min for Hypersil GOLD columns.

Table S1. LC & MS Source Settings for Accucore and Hypersil GOLD columns

Parameter	Hypersil GOLD C4/C18 columns 2.1 x 150 mm, 1.9 µm
Flow rate*	0.4 mL/min
Pos. ion voltage (V)	3500
Sheath gas (arb)	45
Aux gas (arb)	10
Sweep gas (arb)	1
Ion Transfer Tube Temp (°C)	300
Vaporizer Temp (°C)	350

*Solvent A: 0.1% DFA in water. Solvent B: 0.1% DFA in 95% acetonitrile/5% water

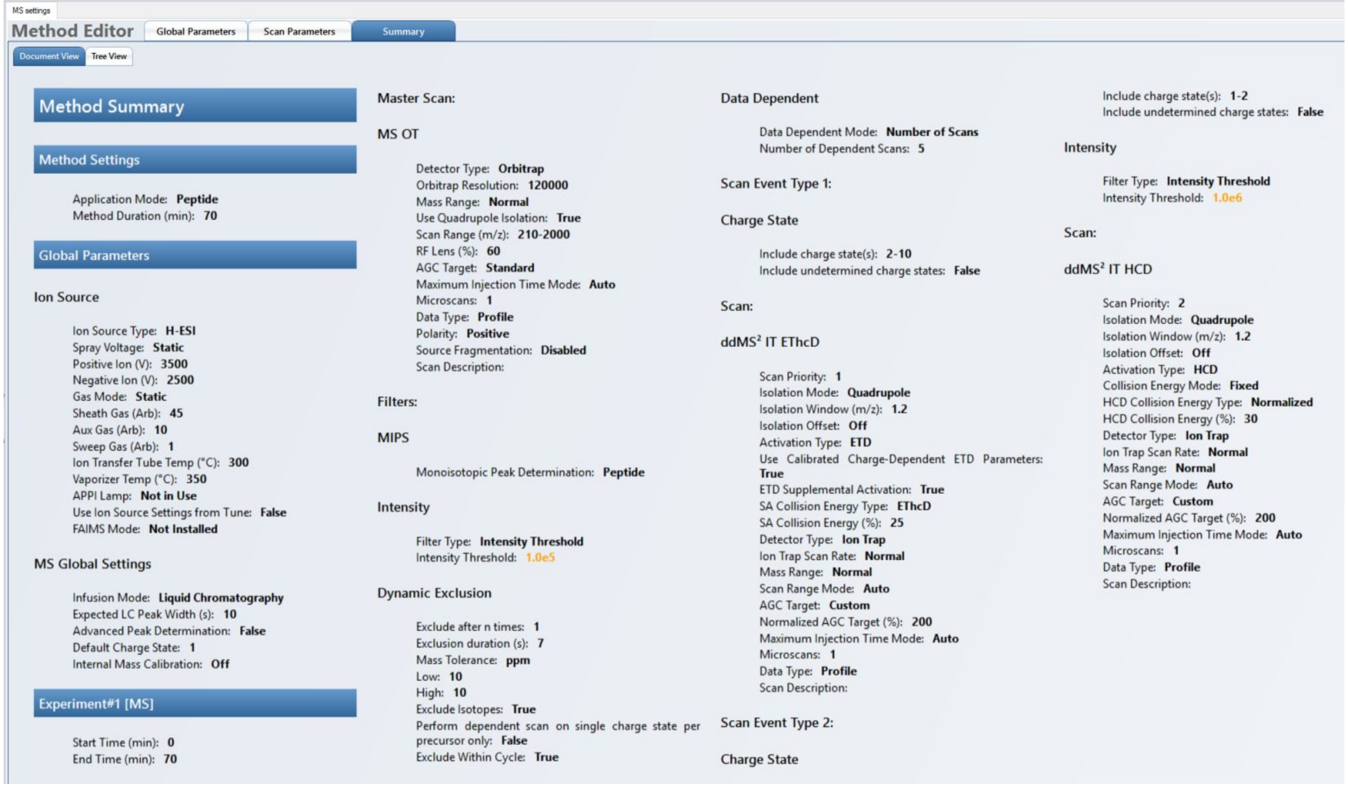

Figure S3. Orbitrap Fusion MS method settings used in the current study.

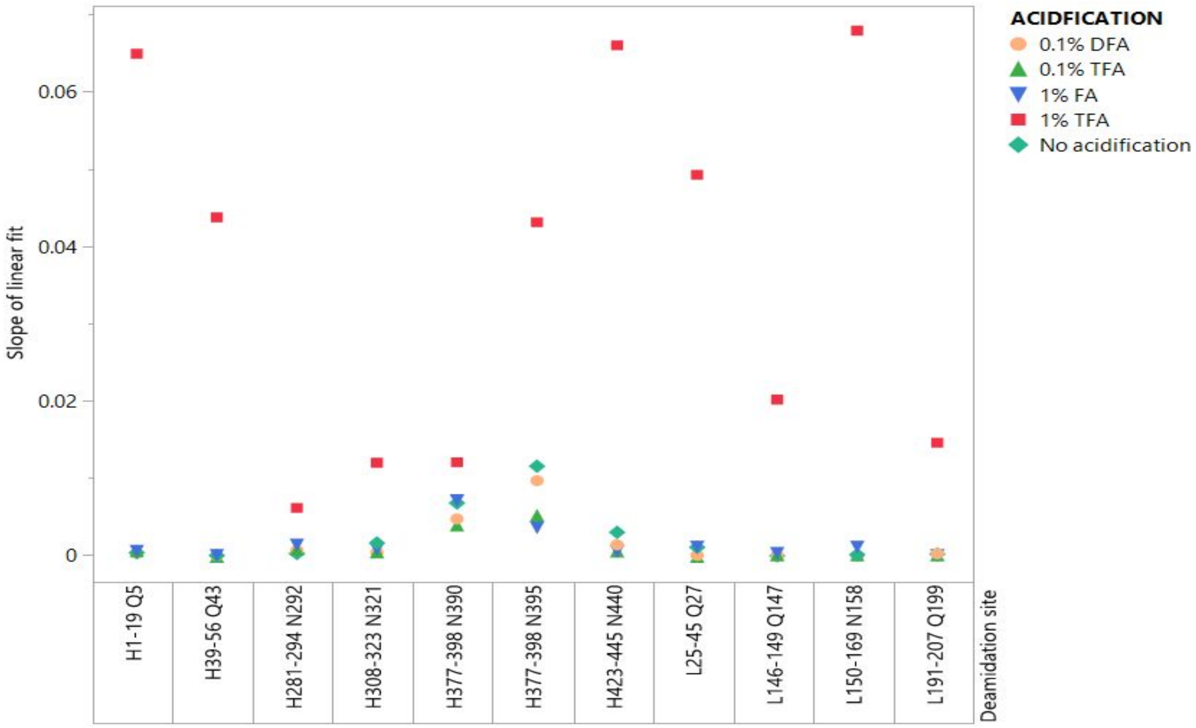

Figure S4. Deamidation levels (extracted ion current ratio) was plotted against time (hrs) in auto sampler and linear regression was performed. The figure shows the determined slope for each deamidation site and acidification condition. A value close to zero indicates no increase in deamidation levels over time in the autosampler. A positive value indicates increasing deamidation level over time in the autosampler. Acidification using 1% TFA consistently gives a higher slope than other acidification conditions, confirming that acid-induced deamidation is taking place over time in the autosampler. H: heavy chain. L: light chain. Numbers refer to amino acid residues.
